# 中国埃克替尼治疗非小细胞肺癌专家共识（2016年版）

**DOI:** 10.3779/j.issn.1009-3419.2016.07.12

**Published:** 2016-07-20

**Authors:** 远凯 石, 燕 孙, 翠敏 丁, 子平 王, 长利 王, 冲 白, 春学 白, 继锋 冯, 晓晴 刘, 方 李, 跃 杨, 永前 束, 密璐 吴, 建行 何, 沂平 张, 树才 张, 公琰 陈, 红鹤 罗, 荣城 罗, 彩存 周, 青松 庞, 兴胜 胡, 宏 赵, 琼 赵, 爱琴 顾, 扬 凌, 诚 黄, 宝惠 韩, 顺昌 焦, 红 简

**Affiliations:** 1 100021 北京，国家癌症中心/北京协和医学院中国医学科学院肿瘤医院内科，抗肿瘤分子靶向药物临床研究北京市重点实验室 National Cancer Center/Cancer Hospital, Chinese Academy of Medical Sciences and Peking Union Medical College, Beijing Key Laboratory of Clinical Study on Anticancer Molecular Targeted Drugs, Beijing 100021, China; 2 050000 石家庄，河北医科大学第四医院 The Fourth Hospital of Hebei Medical University, Shijiazhuang 050000, China; 3 100142 北京，北京大学肿瘤医院 Beijing Cancer Hospital, Beijing 100142, China; 4 300070 天津，天津医科大学肿瘤医院 Tianjin Medical University Cancer Institute and Hospital, Tianjin 300070, China; 5 200433 上海，第二军医大学长海医院 The Second Military Medical University Changhai Hospital, Shanghai 200433, China; 6 200032 上海，复旦大学附属中山医院 Fudan University Zhongshan Hospital, Shanghai 200032, China; 7 210009 南京，江苏省肿瘤医院 Jiangsu Cancer Hospital, Nanjing 210009, China; 8 100071 北京，解放军第307医院 The 307th Hospital of Chinese People's Liberation Army, Beijing 100071, China; 9 100853 北京，解放军总医院 Chinese People's Liberation Army General Hospital, Beijing 100853, China; 10 210029 南京，江苏省人民医院 Jiangsu Province Hospital, Nanjing 210029, China; 11 810000 西宁，青海大学附属医院 Qinghai University Affiliated Hospital, Xining 810000, China; 12 510000 广州，广州医科大学附属第一医院 The First Affiliated Hospital of Guangzhou Medical Unversity, Guangzhou 510000, China; 13 310022 杭州，浙江省肿瘤医院 Zhejiang Cancer Hospital, Hangzhou 310022, China; 14 101149 北京，首都医科大学附属北京市胸科医院 Beijing Chest Hospital, Capital Medical University, Beijing 101149, China; 15 150081 哈尔滨，哈尔滨医科大学附属肿瘤医院 Harbin Medical University Cancer Hospital, Harbin 150081, China; 16 510080 广州，中山大学附属第一医院 The First Affiliated Hospital, Sun Yat-sen University, Guangzhou 510080, China; 17 510515 广州，南方医科大学南方医院 Nanfang Medical University Nanfang Hospital, Guangzhou 510515, China; 18 200433 上海，同济大学附属上海市肺科医院 Tongji University Affiliated Shanghai Pulmonary Hospital, Shanghai 200433, China; 19 310003 杭州，浙江大学附属第一医院 The First Affiliated Hospital, Zhejiang University, Hangzhou 310003, China; 20 200030 上海，上海交通大学附属胸科医院 Shanghai Chest Hospital, Shanghai Jiaotong University, Shanghai 200030, China; 21 213001 常州，常州市第四人民医院 Changzhou Fourth People's Hospital, Changzhou 213001, China; 22 350014 福州，福建省肿瘤医院 Fujian Cancer Hospital, Fuzhou 350014, China

2015年中国肺癌新发病例73.3万，死亡病例61.0万。发病率和死亡率均居恶性肿瘤的首位^[1]^。80%多的肺癌患者为非小细胞肺癌（non-small cell lung cancer, NSCLC），70%左右的患者数就诊时已属晚期，失去了手术治疗的机会。化疗是晚期NSCLC治疗的主要手段，其地位虽然没有发生根本的动摇，但其疗效已达到平台，同时化疗的毒副反应也限制了其广泛的临床应用。近年来，表皮生长因子受体酪氨酸激酶抑制剂（epidermal growth factor receptor-tyrosine kinase inhibitor, EGFR-TKI）由于其确切的疗效、轻微的不良反应和口服给药的便利等特点，突破了传统化疗药物的瓶颈，已经成为晚期NSCLC治疗中不可或缺的重要手段。目前我国已上市的EGFR-TKI药物包括埃克替尼、吉非替尼和厄洛替尼。埃克替尼（商品名：凯美纳）是我国第一个拥有自主知识产权的EGFR-TKI药物，也是全球第三个上市的EGFR-TKI，“小分子靶向抗癌药盐酸埃克替尼开发研究、产业化和推广应用”获得2015年国家科学技术进步奖一等奖。埃克替尼自2011年6月7日被中国国家食品药品监督管理总局（China Food and Drug Administration, CFDA）批准上市，截止到2016年5月31日，已经积累了约10万例NSCLC患者的治疗经验，其中32, 500例患者获得6个月以上的慈善赠药。为进一步规范、指导临床医生更好的使用埃克替尼，更好地为肺癌患者服务，我们组织全国专家，结合我国多个肺癌诊疗规范及治疗指南，于2015年制定了《中国埃克替尼治疗非小细胞肺癌专家共识（2015版）》^[2]^。一年来，埃克替尼新的研究结果不断问世，为了将这些新的研究成果纳入专家共识，中国药学会抗肿瘤药物专业委员会组织专家将原专家共识进行了更新，制定了《中国埃克替尼治疗非小细胞肺癌专家共识（2016年版）》。

## 1 *EGFR*基因敏感突变的晚期NSCLC患者的一线治疗

*EGFR*基因突变状态是晚期NSCLC最重要的疗效预测因子和选择治疗方式的分子指标^[2, 3]^。突变最常见于18-21外显子，其中19外显子缺失突变和21外显子点突变是最常见的*EGFR*基因敏感突变。肺癌突变联盟（Lung Cancer Mutation Consortium, LCMC）的最新研究^[3]^发现，*EGFR*基因敏感突变的晚期NSCLC患者接受EGFR-TKI治疗后的中位生存时间可达到4年。多项研究结果也显示，在非选择的中国NSCLC患者*EGFR*基因敏感突变率约为30%，而肺腺癌患者的敏感突变率可以达到50%^[4, 5]^，不吸烟的腺癌患者甚至可高达60%-70%，肺鳞癌患者也仍有10%左右的*EGFR*突变率^[6, 7]^，因此建议临床上对已经明确NSCLC的病理诊断、并且不能进行手术的晚期患者，在治疗开始前要常规进行*EGFR*基因突变检测。近年来多项一线随机Ⅲ期临床研究（包括IPASS、NEJ002、WJTOG3405、OPTIMAL、EURTAC、LUXLUNG3、LUXLUNG6）^[8-14]^结果显示，EGFR-TKI一线治疗*EGFR*基因敏感突变晚期NSCLC患者的无进展生存期（progression free survival, PFS）可以达到9.5个月-13.7个月，远高于传统一线化疗方案的4.6个月-6.9个月，总有效率也同样显示EGFR-TKI明显优于传统化疗（EGFR-TKI和传统化疗分别是58%-84%和15%-47%）。并且所有研究均显示EGFR-TKI的不良反应更轻，尤其是血液学毒性，患者耐受性更好，生活质量显著改善。埃克替尼上市后开展了IV期临床研究^[15]^，自2011年8月至2012年8月共入组了6, 087例接受埃克替尼治疗的晚期NSCLC患者，其中989例患者接受了*EGFR*基因突变检测，738例*EGFR*基因敏感突变患者的客观缓解率（objective response rate, ORR）和疾病控制率（disease control rate, DCR）分别是49.2%和92.3%。其中接受埃克替尼一线治疗的144例患者，ORR和DCR分别是56.3%和95.1%。另一项研究^[16]^回顾性分析了2009年3月-2012年1月北京胸科医院收治的59例接受埃克替尼治疗的晚期NSCLC患者的疗效，其中20例患者接受埃克替尼一线治疗，部分缓解（partial response, PR）8例，疾病稳定（stable disease, SD）7例，疾病进展（progressive disease, PD）5例。20例埃克替尼一线治疗患者中有8例存在*EGFR*基因敏感突变，19外显子缺失突变的5例均获得PR，21外显子点突变的3例患者中，PR 1例，SD 1例，PD 1例。因此，2013年、2014年及2015年的医学信息管理系统（Medical Information Management System, MIMS）恶性肿瘤用药指南^[17]^、《中国原发性肺癌诊疗规范(2015年版)》^[18]^和《中国晚期原发性肺癌诊治专家共识（2016年版）^[19]^均推荐埃克替尼作为*EGFR*基因敏感突变晚期NSCLC患者的一线治疗药物。在2016年美国临床肿瘤学会（American Society of Clinical Oncology, ASCO）上公布的埃克替尼对比化疗一线治疗*EGFR*基因敏感突变的晚期肺腺癌患者的CONVINCE研究结果显示^[20]^，埃克替尼显著改善患者PFS（埃克替尼组296天*vs*化疗组219天，HR 0.67，*P*=0.008）和ORR（埃克替尼组64.8% *vs*化疗组33.8%，*P* < 0.001），埃克替尼组患者的不良反应发生率明显低于化疗组（70.3% *vs* 88.3%, *P* < 0.001），埃克替尼组常见的不良反应是转氨酶升高（29.1%）、皮疹（17.6%）和腹泻（9.5%），化疗组与既往报道的相似。目前埃克替尼正在开展多项针对*EGFR*基因敏感突变晚期NSCLC患者一线治疗的临床研究，包括一线治疗脑转移的BRAIN研究（NCT01724801）和一线治疗老年突变患者的研究（NCT01646450）等。2014年11月埃克替尼获得CFDA新治疗适应症批准，用于一线治疗*EGFR*基因突变晚期NSCLC [批件号：2014B02155]，这也是继吉非替尼之后第二在中国获得该适应症的EGFR-TKI药物。

## 2 晚期NSCLC的维持治疗

全球多项一线化疗后EGFR-TKI维持治疗研究的结果均显示，*EGFR*基因敏感突变的晚期NSCLC患者可以从EGFR-TKI维持治疗中获益^[21-23]^。一项2009年3月至2012年1月北京胸科医院收治的59例接受埃克替尼治疗的晚期NSCLC患者的回顾性研究^[16]^中有2例*EGFR*基因敏感突变患者在一线化疗后接受了埃克替尼维持治疗，疗效均达PR。埃克替尼目前虽然没有维持治疗的前瞻性研究，但是可以进行这方面的探索。

## 3 晚期NSCLC二、三线治疗

ISEL、INTEREST、TITAN、BR21等多项临床研究以及荟萃（*meta*）分析^[24-28]^的结果均显示，对于未经选择的亚裔复发晚期NSCLC患者，EGFR-TKI能显著降低疾病进展风险，提高肿瘤的客观缓解率，总体疗效与标准的二线化疗相当，但耐受性更好。因此奠定了EGFR-TKI在晚期NSCLC二、三线治疗的地位。ICOGEN研究^[29]^是我国开展的一项非劣效性Ⅲ期临床试验，比较了埃克替尼与吉非替尼治疗未经选择的复治的晚期NSCLC患者的疗效和安全性。该研究也是全球第一项两个EGFR-TKI药物之间头对头比较的Ⅲ期临床研究。结果显示埃克替尼的疗效不劣于吉非替尼，主要终点指标PFS埃克替尼组4.6个月、吉非替尼组3.4个月；药物相关不良事件埃克替尼组61%、吉非替尼组70%（*P*=0.046），常见不良反应腹泻的发生率埃克替尼组较吉非替尼组显著降低（埃克替尼组19%、吉非替尼组28%，*P*=0.033）。研究中对能收集到肺癌组织标本的患者进行了*EGFR*基因敏感突变状态的检测，发现无论是*EGFR*基因突变型或野生型患者，吉非替尼与埃克替尼的PFS、OS均无差别，PFS：埃克替尼组7.8个月、吉非替尼组5.3个月，OS：埃克替尼组20.9个月、吉非替尼组20.2个月；吉非替尼与埃克替尼对*EGFR*基因敏感突变型患者的PFS、OS均优于野生型患者（*P* < 0.001）。2013年、2014年《MIMS恶性肿瘤用药指南》^[17]^、2014年版《临床路径治疗药物释义·肿瘤疾病分册》^[30]^、2015年版《临床路径治疗药物释义·肿瘤疾病分册》^[31]^、2013年-2015年版《中国表皮生长因子受体基因敏感突变和间变淋巴瘤激酶融合基因阳性非小细胞肺癌诊断治疗指南》^[32-34]^、《中国原发性肺癌诊疗规范(2015年版)》^[18]^和《中国晚期原发性肺癌诊治专家共识(2016年版) 》^[19]^均推荐埃克替尼用于晚期NSCLC患者的二、三线治疗。

## 4 EGFR-TKI新辅助和辅助治疗

EGFR-TKI的新辅助和辅助治疗目前国际上没有明确结论，埃克替尼等EGFR-TKI的多项研究正在进行中（NCT01929200, NCT01843647）。这些研究结果将回答EGFR-TKI是否可以使*EGFR*基因敏感突变的Ⅱ期-Ⅲa期肺腺癌患者从靶向治疗中获益。

## 5 脑转移NSCLC治疗

埃克替尼治疗肺腺癌脑转移患者同样取得了很好的疗效。一项回顾性研究^[35]^对埃克替尼治疗28例就诊于中国医学科学院肿瘤医院的肺腺癌脑转移患者进行了分析。其中12例患者*EGFR*敏感突变，14例患者*EGFR*无突变，2例患者*EGFR*突变状态不明。所有患者ORR为67.8%，DCR为96.4%。*EGFR*敏感突变的患者ORR为91.7%，DCR为100%。*EGFR*无突变或突变状态不明的患者ORR为50%，DCR为93.8%。

总之，埃克替尼使我国有了国产的EGFR-TKI，使我国NSCLC患者有了新的治疗选择，专家委员会将随着研究结果的不断增多，适时的更新本共识。
